# β‐1,4‐galactosyltransferase 1 inhibitors modulate cellular N‐glycan profiles

**DOI:** 10.1002/btpr.88522

**Published:** 2026-05-21

**Authors:** Jaka Kranjc, Stane Pajk, Marko Anderluh, Anja Pišlar, Matjaž Brinc

**Affiliations:** ^1^ Institute for Pharmacy, Faculty of Pharmacy University of Ljubljana Ljubljana Slovenia; ^2^ Department of Pharmaceutical Chemistry Faculty of Pharmacy, University of Ljubljana Ljubljana Slovenia; ^3^ Department of Pharmaceutical Biology Faculty of Pharmacy, University of Ljubljana Ljubljana Slovenia; ^4^ Bioprocess Development, Technical Development Biologics Novartis Technical Research & Development, Novartis LLC Mengeš Slovenia

**Keywords:** bioprocess, Chinese hamster ovary, galactosyltransferase, glycosylation, inhibitors

## Abstract

N‐glycosylation is a key post‐translational modification of proteins influencing their physicochemical properties as well as biological activity. This study investigates the *in vivo* activity of four β‐1,4‐galactosyltransferase 1 inhibitors in cell cultures producing recombinant IgG antibodies. Inhibitors, previously identified as potent inhibitors in on‐target assay, were evaluated in fed‐batch bioprocesses. Dose‐dependent effects on cell culture performance were assessed following compound addition. N‐glycan profiles were analyzed using InstantPC labeling and UHPLC‐FLD, while intracellular compound concentrations were quantified via LC–MS/MS following time‐resolved sampling of treated cell lysates. Bioprocess parameters, including cell growth, viability, and productivity, were monitored throughout cultivation. The study revealed divergent behaviors between on‐target potency and *in vivo* activity, underscoring the importance of cellular permeability, metabolic stability, and off‐target effects of potential inhibitors used in cell culture. These findings provide new insights into the challenges of glycosylation pathway modulation and inform strategies for future glycoengineering tool development.

## Graphical abstract

Graphical representation of the study workflow used to investigate the effects of known in vitro galactosyltransferase inhibitors on cell culture performance and recombinant IgG galactosylation.

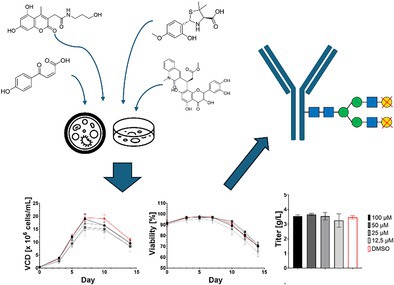

## 1 INTRODUCTION

Glycosylation is one of the most common and complex post‐translational modifications of proteins, significantly influencing their stability, activity, and therapeutic potential. Among the various glycosylation pathways, N‐glycosylation plays a critical role in the folding, secretion, and biological function of recombinant proteins,^1^ including biopharmaceuticals such as monoclonal antibodies.^2^ Modifying glycan structures through specific enzymatic inhibition is a powerful strategy for controlling protein quality and enhancing the efficacy of biopharmaceuticals.^3^ However, developing selective and cellularly effective inhibitors targeting glycosylation enzymes remains a significant challenge.^4^

β‐1,4‐galactosyltransferase 1 (B4GALT1) is a key enzyme in the Golgi glycosylation pathway, responsible for adding galactose to N‐linked glycans.^5^ Modulation of B4GALT1 activity can influence the galactosylation of glycoproteins,^6^ a modification that affects protein stability,^1^ immune recognition, and receptor binding.^7^ For example, reducing antibody galactosylation can enhance their binding to certain receptors, potentially improving therapeutic outcomes.^8^ As a result, B4GALT1 has emerged as a promising target for the development of selective inhibitors to fine‐tune glycosylation patterns in biopharmaceutical production. However, despite significant research efforts, identifying effective B4GALT1 inhibitors that function in cells remains a major challenge. Many inhibitors that show promising potency in on‐target assays fail to exhibit their effects in cellular systems due to poor cell membrane permeability.^9^, ^10^, ^11^

In this study, we investigated the efficacy of four B4GALT1 inhibitors in cell culture previously identified as potent B4GALT1 inhibitors in an enzyme inhibitory assay. The primary aim was to evaluate their impact on galactosylation in CHO cell cultures producing recombinant IgG antibodies, using both 24‐deepwell plate and Ambr15 bioreactor cultivation systems. To gain insights into the mode of action of the investigated compounds, their cellular permeability was assessed using an LC/MS‐based method. Through these experiments, we aimed to elucidate the challenges of translating on‐target inhibitory activity into functional outcomes and identify compounds capable of specifically modulating glycosylation with minimal impact on overall cell culture performance.

## 2 MATERIALS AND METHODS

### 2.1 Cell cultivation and bioprocess conditions

A CHO cell line, derived from an internally developed CHO K1 strain producing an IgG antibody, was utilized for the study. Following thawing, cells were cultured in shake flasks (Corning) using a proprietary, chemically defined medium provided by Irvine Scientific. The cells were passaged at least four times to ensure adequate recovery and prepare for inoculation into the Ambr15 bioreactor system and deep‐well plates (DWPs) at a target viable cell density (VCD) of 0.4 × 10^6^ viable cells/mL. Cultures were grown in ISF1 X incubator shakers (Kuhner) at 36.5°C with 10% CO_2_, a shaking speed of 200 rpm, and a shaking radius of 12.5 mm to maintain optimal growth conditions.

Cells cultivated in 24‐well DWPs and Ambr15 bioreactors were used to evaluate four novel B4GALT1 inhibitors. Cells were cultivated according to an in‐house proprietary fed‐batch platform. The process conditions, intra‐process controls, and process harvest were performed according to the same protocol as already described in our previous work.^12^ Table 1 summarizes the corresponding process parameter settings. Clarified bioprocess harvest from day 14 was sent for N‐glycan analysis.

**TABLE 1:** Summary of process parameters for cell cultures grown in DWPs and Ambr15.

Process parameter	DWP	Ambr15
Seeding VCD (× 10^6^ viable cells/mL)	0.4	
Working volume (mL)	3	14.5
Temperature at start	36.5°C	
Agitation rate (rpm)	300	700
pH setpoint	/	7.1 ± 0.1
CO_2_ gassing	10%	On demand
0.5 M NaOH addition	/	On demand
Feeding start (day)	4	2
Day of temperature shift	4	5
Temperature after shift	33°C	
Duration of cultivation (day)	14	

On the day of the temperature shift, all cell cultures were treated with either a stock solution of the investigated compounds dissolved in DMSO or with pure DMSO as a control. The chemical structures of the compounds used are presented in Table 2 below. For DWP cultures, 30 μL of the DMSO solutions were added, while for Ambr15 cultures, 145 μL of DMSO solutions were used. In all cases, the final percentage of DMSO in culture was 1% (v/v). The highest concentration of compound stock solutions was 10 mM, resulting in a final concentration of 100 μM upon addition to the cell culture. For each compound, four stock solutions were prepared using DMSO at concentrations 10, 5, 2.5, and 1.25 mM. After addition to cell cultures, the final concentrations of compounds were 100, 50, 25, and 12.5 μM, respectively. Each condition was tested in triplicate. The arrangement of conditions within the Ambr15 system was randomized.

**TABLE 2:** Structures of compounds used for permeability testing and cell culture addition.

Compound No.	Vendor and compound code	Structural formula	IC_50_ value (μM)^a^
**6**	Vitas m‐labs—STL543148	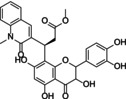	6.2 ± 2.1^13^
**8**	Key Organics—MS‐11121	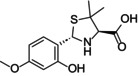	43.0 ± 3.7^13^
**12**	Key Organics—24849‐48‐7	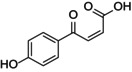	13.1 ± 8.2^13^
**28**	Vitas m‐labs—STL518381	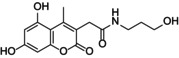	8.0 ± 2.4^13^

*Note*: Compound numbers are preserved from our previously published work investigating these compounds.^13^

^a^ B4GALT1 inhibition constant obtained by using UDP‐Glo assay.

For the DWP cultures, a single plate was used that included all investigated conditions for compound **6**, and control runs with pure DMSO or water for injection (WFI). One Ambr15 campaign was performed with 48 bioreactors, 39 of which received either an addition of stock solutions of compounds **8**, **12**, and **28**, pure DMSO, or sterile WFI. The remaining nine bioreactors served as backups and received no additions of solutions or solvents.

### 2.2 Cell membrane permeability and preparation of cell lysates

To assess the cell membrane permeability of the compounds, we utilized a CHO cell line derived from an internally developed CHO‐K1 line grown in shake flasks as described in Section 2.1. Following four passages in shake flasks, an aliquot of the cell culture was diluted with fresh medium in a shake flask to achieve a VCD of 5 × 10^6^ viable cells/mL. The culture was then divided into five separate 10 mL aliquots, each placed in 50 mL centrifuge tubes. Each aliquot, except for the control, was treated with 5 μL of a 50 mM compound stock solution in pure DMSO for the times indicated. The control aliquot was treated with 5 μL of pure DMSO.

The aliquots were incubated in incubator shakers under growth conditions as described in Section 2.1. Samples (2 mL each) were collected at 15, 30, 60, and 120 min. Each sample was centrifuged at 10,000×*g* for 10 min at room temperature, and the supernatant was discarded. The resulting cell pellets were resuspended in 2 mL of phosphate buffered saline (PBS, pH 7.4) to remove any compounds bound to the cell membrane. After another round of centrifugation under the same conditions, the supernatant was again discarded, and the pellets were resuspended in 1.5 mL of HEPES lysis buffer (50 mM HEPES, pH 6.5, 150 mM NaCl, 1 mM EDTA, 0.25% Triton X‐100) and incubated for 20 min on ice. Following overnight freezing at −70°C, samples were thawed and centrifuged at 10,000×*g* for 10 min and filtered through a 0.2 μm Millipore Millex filter (cat. No. SLGV033RB). The filtrate was diluted 4‐fold with cold acetonitrile to precipitate any remaining proteins. After a 20‐min incubation, samples were centrifuged again at 30,000×*g* for 30 min at room temperature. The resulting supernatant was transferred into cryovials (Corning, cat. No. # 431386) and stored at −20°C until subsequent analysis by LC/MS.

The LC–MS/MS method was tested for accuracy, precision, linear range, lower limit of quantification, and absolute matrix effect. The calibration samples were prepared in a water/acetonitrile mixture (4/1). Eight‐point matrix calibration curve was prepared with concentration range from 20 μM to 200 μM. The measured responses across the whole range were linear with acceptable accuracy (±15% bias) and precision (15% RSD) and had a good correlation (*R* ^2^ above 0.99).

Quality control samples were prepared the same way as for the calibration at 60 μM. Accuracy was determined by comparing the measured concentrations of the quality control samples to their true values and expressed as bias (%). Method repeatability was determined by calculating the RSD from the separately prepared and analyzed quality control samples. Accuracy and precision were determined to be 1.0 and 2.1, respectively.

#### 2.2.1 LC/MS analysis of cell lysates

Chromatographic separation was performed on a Vanquish Flex UHPLC system (Thermo Fisher Scientific) equipped with a Kinetex PS C18 column (100 mm × 2.1 mm, 2.6 μm particle size; Phenomenex). The column temperature was maintained at 40°C and the autosampler was kept at 5°C. The mobile phases composition and gradient elution programs were optimized individually for each compound as follows in Table 3.

**TABLE 3:** Gradient elution programs for LC/MS analysis of each compound.

Compound	Gradient elution	Flow rate (mL/min)
**6**	0–4.0 min, 20%–65% B; 4.0–4.1 min, 65%–100% B; 4.1–5.0 min, 100% B; 5.0–5.1 min, 100%–20% B; 5.1–7.0 min, 20% B	0.50
**8**	0–3.5 min, 5%–35% B; 3.5–4.0 min, 35%–100% B; 4.0–5.0 min, 100% B; 5.0–5.1 min, 100%–5% B; 5.1–7.0 min, 5% B	0.50
**12**	0–1.0 min, 5% B; 1.0–7.0 min, 5%–100% B; 7.0–8.0 min, 100% B; 8.0–8.1 min, 100%–5% B; 8.1–10.0 min, 5% B	0.50
**28**	0–1.0 min, 5% B; 1.0–7.0 min, 5%–100% B; 7.0–8.0 min, 100% B; 8.0–8.1 min, 100%–5% B; 8.1–10.0 min, 5% B	0.40

*Note*: The mobile phases consisted of water/acetonitrile/formic acid (950:50:1, v/v/v) as solvent A and water/acetonitrile/formic acid (50:950:1, v/v/v) as solvent B.

MS/MS analysis was carried out on a TSQ Fortis triple quadrupole mass spectrometer (Thermo Fisher Scientific) equipped with a heated electrospray ionization (H‐ESI) source. Single‐reaction monitoring (SRM) mode was used for ion detection. Detailed MS/MS parameters and compound‐specific chromatographic conditions are provided in Table S1.

Instrument control, data acquisition, and quantification were performed using Xcalibur software (Thermo Fisher Scientific).

#### 2.2.2 N‐glycan analysis

Samples were prepared for N‐glycan analysis using a commercially available kit produced by Agilent. The kit contained an Agilent AdvanceBio Gly‐X deglycosylation module, InstantPC labeling module, and InstantPC, which were used following the manufacturer's protocol.^14^ The procedure used was identical to that described in our previously published work.^12^

## 3 RESULTS AND DISCUSSION

### 3.1 Effects of inhibitors on cell growth, viability, and antibody productivity

**FIGURE 1:**
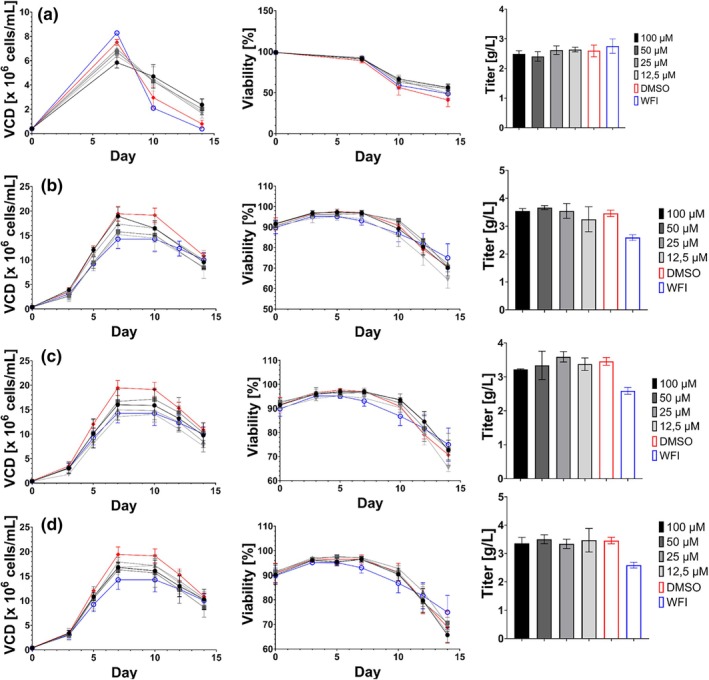
Viable cell density (VCD) profiles, viability profiles and titers. Cells cultured in Ambr15 or deep well plates were supplemented with compounds at different concentrations (12.5–100 μM) on day 5. For control DMSO and WFI were used at the same final concentration. Red and blue lines and bars represent controls (DMSO and WFI, respectively), and different shades of gray compound treatment (a: Bioprocesses with compound **6** addition, b: Bioprocesses with compound **8** addition, c: Bioprocesses with compound **12** addition, d: Bioprocesses with compound **28** addition). Standard deviation intervals are indicated by error bar and are mean of three parallel runs.

To assess the inhibitory activity of selected B4GALT1 inhibitors in a bioprocess context, cell culture performance under compound treatment was systematically evaluated. Cells were cultivated in two setups, one being 24‐well DWP and the other being Ambr15 bioreactor system. When the cells reached a predetermined VCD value, the temperature setpoint was shifted to 33°C. This adjustment was performed on day 5 of cultivation for all cell cultures. On the same day, DMSO solutions of the investigated compounds or control solutions (DMSO or WFI) were added into the cell cultures. In Figure 1, the growth curves for cultures with added inhibitors as well as the control conditions are shown. In addition to the compounds mentioned in this section, the effect of Alendronate (a known B4GALT1 inhibitor) was tested in cell culture (see Figures S1 and S2).

The VCD curves displayed in the first column of graphs in Figure 1 show that the cells grew as expected of a fed‐batch culture. All cultures reached the peak VCD on day 7, after which the VCD values gradually declined. Cultures treated with compounds **6**, **12**, and **28** exhibited slightly lower peak VCDs compared to the DMSO control, suggesting a potential inhibitory effect on cell proliferation. However, this effect is likely an artifact, as several error bars overlap with the control processes. In contrast, cultures exposed to the highest concentration of **8** reached a peak VCD comparable to the DMSO control but showed a more rapid decline, thereafter further indicating a possible negative impact on cell growth. For cultures grown in the Ambr15, the addition of DMSO increased the peak VCD compared to WFI control. However, on day 14, most cultures had comparable VCD measurements, suggesting that while the tested compounds may transiently impair cell growth, their effects were not sustained for the full duration of the fed‐batch process. In DWP, the WFI control reached a higher peak VCD than the processes with added DMSO but also dropped to comparable levels by the end of the process. Notably, the VCD of the DMSO control dropped below that of the cultures treated with compound **6**. This is unusual, as none of the other compounds displayed this phenomenon, nor is it in line with previous experience with this cell line or bioprocess. Since **6** conditions were grown in DWP format, it is plausible that the observed anomaly is linked to format‐specific variables. One hypothesis is that the DMSO control wells were located at the plate edges, potentially exposing them to altered thermal conditions and evaporation that may have impacted cell growth.

Similar to the VCD trends/profiles, all conditions show a decline of cell viability towards the end of the process. Although the drop in viability may appear substantial, it is typical for the cell line used and an endpoint viability of 50% remains within acceptable limits. There is a peculiar occurrence visible for **8** where the lowest concentration of the compound addition causes lower viability throughout the process. While the corresponding curve (light gray line) appears distinct in the viability plot, the relatively large standard deviation error bars overlap with those of other conditions, suggesting the difference may not be statistically significant. This indicates that factors other than **8** addition may have contributed to the observed lower viability. Due to the complexity of biological expression systems and bioprocesses themselves, pinpointing these factors is not possible. A similar occurrence is visible on the viability graph for **12** where the condition of the lowest compound concentration also deviates from the others; however, this is apparent only on the final day of cultivation.

Final examination of the bar graphs showing relative antibody titer measurements measured on the last day of cultivation, it can be seen that the additions of the investigated compounds had no negative effects on productivity. Some conditions, such as the 12.5 μM of **8** and **28**, or 50 μM of **12**, exhibit relatively broad error bars. Again, this variability can be attributed to external factors, as already discussed above. In Ambr15 cultures, addition of DMSO solutions caused an increase in titer compared to WFI control. Interestingly, in DWP culture, this was not the case, with the most probable cause again being format‐specific variables.

### 3.2 Analysis of N‐glycan profiles following B4GALT1 inhibitors treatment

**FIGURE 2:**
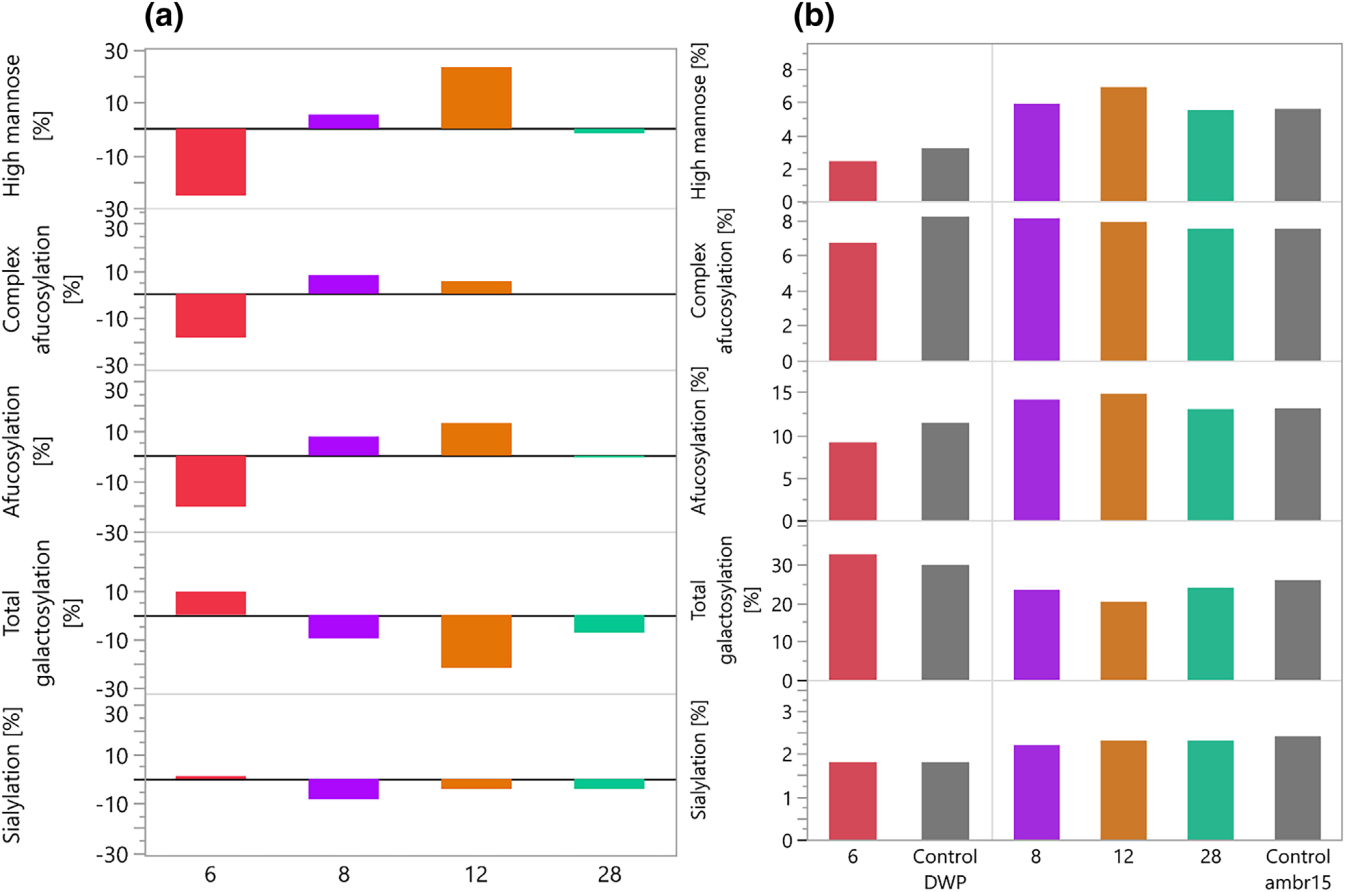
Impact of B4GALT1 inhibitors on glycan group profiles in cell cultures. (a) Relative change of glycan group measurements for selected cell cultures treated with B4GALT1 inhibitors (100 μM) compared to control (DMSO). (b) Raw measured values of glycan group measurements. Gray bars represent the values measured for control bioprocesses with added DMSO for each cultivation system.

To evaluate the impact of the investigated B4GALT1 inhibitors on protein glycosylation, N‐glycan profiles of recombinant IgG produced under different treatment conditions were analyzed. The results of the N‐glycan analysis are shown in Figure 2. No error bars are shown, as only one sample per condition was analyzed. Bioprocesses were performed using a standardized platform process with extensive prior data (data not shown), and a validated analytical method was used for the analysis. The results should therefore be interpreted as trend‐indicating observations rather than statistically demonstrated differences. All the identified glycans were assigned into six groups based on the sugar molecules comprising the glycan.

Examination of the high‐mannose group reveals variable effects across the different compounds investigated. **6** led to a reduction in high‐mannose glycan species, suggesting an enhancement in glycan maturation. Although the absolute reduction in high‐mannose species was less than 1%, the relative change was 25%, leading us to believe this effect is not due to standard bioprocess variability. In contrast, **12** and **8** resulted in increased high‐mannose abundance, indicating a shift towards less mature glycan structures. **12** had a strong effect on high‐mannose species inversely comparable to **6**, while **8** was much weaker. **28** caused less than a 0.5% relative reduction of high‐mannose species, which is considered to be negligible. These observations imply that the compounds modulate the glycosylation pathway, either by promoting or hindering glycan processing. Inhibitors can create bottlenecks in glycosylation pathways, causing accumulation of glycan species before the affected enzymatic reactions that are inhibited. Corresponding shifts in the relative abundance of other glycan species would be expected as a consequence of these changes.

To accurately assess the effect of compounds on fucosylation pathways we defined two distinct groups of afucosylated glycans. The “Afucosylation” group includes both high‐mannose glycans and more mature glycans lacking fucose, while the “Complex afucosylation” group consists exclusively of mature glycans without fucose. This distinction allows us to identify through which enzyme interactions the investigated compounds affect the addition of fucose to the growing glycan chain. Notably, **6** is the only compound that reduces complex afucosylation, whereas the other compounds either have no effect or increase complex afucosylation slightly. This observation suggests that **6** has off‐target effects that cause an increase in fucosylation.

All of the compounds investigated affected galactosylation more strongly than any other glycan group. The B4GALT1 enzyme is directly responsible for the addition of galactose residues during glycan maturation, so inhibition of its activity was expected to produce measurable changes in galactosylation levels. However, only three compounds caused a reduction in galactosylation. **12** had the strongest effect, with a reduction of 5.6%, followed by **8** with a reduction of 2.5%, and **28**, which caused the reduction of galactosylation to 1.9%.

**6** unexpectedly increased total galactosylation by almost 3%. Among the four potential inhibitors, it had the lowest measured IC_50_ towards B4GALT1,^13^ leading us to expect a pronounced reduction in galactosylation. This discrepancy suggests that the compound may not effectively reach the target enzyme, possibly due to limited membrane permeability. Alternatively, **6** could exert off‐target effects on enzymes involved in the galactose synthesis pathway or on galactose‐specific transporters located on the Golgi apparatus membrane. Another hypothesis is that the compound is metabolized intracellularly, producing an active metabolite that affects the glycosylation pathway enzymes or substrate availability.

In line with the more pronounced effects observed on galactosylation and high‐mannose structures, the impact of the investigated compounds on sialylated glycan species was relatively weak. Sialylation occurs at the terminal end of the glycosylation pathway and depends on prior processing steps such as galactosylation. Therefore, substantial changes in sialylation would typically be expected only if upstream modifications were strongly altered. While compounds **8**, **12**, and **28** reduced the total galactosylation, there was still enough galactosylated proteins to enable the synthesis of sialylated glycoforms.

Based on these results, **12** appears to be the most effective small molecule tool for reducing galactosylation where the effect on other glycan groups is not critical. However, as it raises the content of high‐mannose as well as afucosylated species, it may not be suitable for use in every bioprocess. In cases where a highly specific effect on galactosylation is required, **28** stands out. Although it is the weakest inhibitor of galactosylation, it minimally impacts the other glycan groups, making it potentially useful for applications demanding selectivity.

### 3.3 Intracellular uptake and metabolic stability of B4GALT1 inhibitors

**FIGURE 3:**
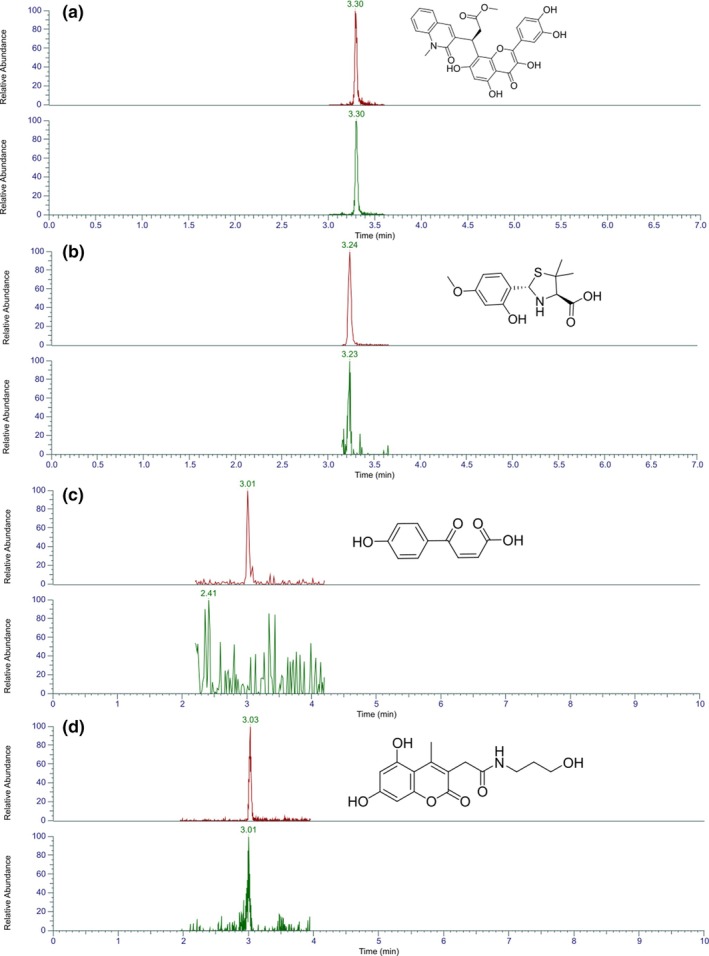
Representative chromatograms of the investigated compound lysates. The red chromatograms show standard solution measurements, while the green chromatograms show the measured samples. (a) Compound **6** standard solution at 20 μM (red) and sample after 15 min of incubation (green). (b) Of compound **8** standard solution at 10 μM (red) and sample after 120 min of incubation (green). (c) Compound **12** standard solution at 50 μM (red) and sample after 15 min of incubation (green). (d) Compound **28** standard solution at 10 μM (red) and sample after 60 min of incubation (green). Chromatograms for all time points can be found in Figures S3–S6.

To better understand the observed differences in compound efficacy, the intracellular uptake of each compound was evaluated. After cell lysates from cultures treated with the investigated compounds were collected at four different time points (15, 30, 60, and 120 min), the content of the compounds permeating into the cells was analyzed using an LC–MS/MS method. The results of these measurements are shown in Figure 3 below. Among all the compounds investigated, only **6** was clearly detectable in the prepared cell lysates.

The lower limit of quantification for **6** was set at the lowest calibration point (20 μM), where the method maintained acceptable accuracy (4% bias). The absolute matrix effect was calculated as a ratio between the peak area response from the control samples, that is, solution of analyte in water/acetonitrile mixture (4/1), and the peak area response from samples prepared with blank cell lysate and spiked with analyte. The absolute matrix effect was determined to be 101.5%. Table 4 below shows the measured concentrations of **6** at different time points.

**TABLE 4:** Measured concentration of **6** in lysate samples at different time points.

Time (min)	Concentration (μM)
15	83
30	56
60	29
120	14

As shown in Table 4, the concentration of **6** detected in the cell lysate decreases over time. This trend may indicate that the compound is metabolized intracellularly as previously hypothesized. The breakdown of **6** by intracellular enzymes could also explain the unexpected effect this compound had on the N‐glycosylation pattern of the recombinant IgG antibody.

For the remaining compounds, no clear signal was detected in the lysate samples at any of the time points analyzed. This result is surprising, as all compounds affected the glycosylation of the produced IgG antibody in the expected manner, namely by reducing galactosylation following their addition to the cell culture. One possible explanation is that compounds **8**, **12**, and **28** do not permeate through the cell membrane efficiently. They may however permeate membranes to a lesser extent that would allow measurable effect on galactosylation, especially if cells are exposed to the compounds for several days as they were in our bioprocess experiment. Also, they could interact with membrane receptors or membrane‐associated signaling molecules, triggering intracellular signaling cascades that ultimately modulate the glycosylation network. However, the specific mechanisms by which such signaling events could influence glycosylation remain unknown and warrant further investigation. An alternative possibility, albeit less likely, is that all of these compounds are taken up by cells but are metabolized more rapidly than **6**. If their intracellular degradation occurs quickly enough, even the earliest time points may have been too late to detect the intact compounds.

Upon examining the chromatograms in Figure 3, an interesting pattern can be seen for **8** and **28**. The peaks between control and sample chromatograms overlap, but the sample chromatograms have much larger noise in the surroundings of the peak. This observation may suggest that the compounds undergo intracellular metabolism, resulting in the formation of various metabolites that interfere with the detection of the parent compound and contribute to the observed chromatographic noise. These findings support the hypothesis that metabolic instability may be a contributing factor to the low detectability of certain compounds in the intracellular environment.

## 4 CONCLUSION

The development of effective and specific inhibitors targeting glycosylation enzymes remains a significant challenge in both glycobiology and biopharmaceutical process development. In this study, we evaluated the *in vivo* activity and potential mechanisms of action of four β‐1,4‐galactosyltransferase 1 (B4GALT1) inhibitors previously identified as reasonably potent on‐target candidates. While three compounds (**8**, **12**, and **28**) successfully reduced galactosylation levels in recombinant IgG produced by CHO cells, **6** unexpectedly increased galactosylation despite its strongest on‐target inhibitory profile. Permeability assessment revealed that **6** was the only compound detected within the cells, suggesting intracellular metabolism may alter its activity or specificity. These results emphasize the complexity of translating findings from early biochemical assays into functional outcomes in cellular systems and reinforce the need to consider membrane permeability, metabolic stability, and potential off‐target effects in early‐stage inhibitor characterization.

Interestingly, compounds **8**, **12**, and **28** consistently exerted inhibitory effects on galactosylation although undetected in cell lysates. This raises the possibility of extracellular or membrane‐associated modes of action, potentially involving signaling pathways that modulate glycosylation without direct intracellular access. These findings point to an underexplored aspect of glycoengineering: the indirect regulation of glycan biosynthesis through membrane‐bound or extracellular mechanisms. Further mechanistic studies will be essential to elucidate these pathways and to determine their potential utility in modulating glycosylation. From a bioprocessing perspective, all tested compounds exhibited only transient or minor effects on cell growth, viability, and antibody productivity across both 24‐well plate and Ambr15 bioreactor platforms. Glycan profiling revealed that, although galactosylation was the most consistently affected, other glycan groups, including high‐mannose and afucosylated species, were also modulated to varying extents. **12** was the most effective at reducing galactosylation, but it also increased high‐mannose and afucosylated glycans, limiting its applicability in contexts requiring high glycan maturity. In contrast, **28**, while less potent in galactosylation inhibition, offered the most selective effect with minimal impact on other glycan species. This makes it a promising candidate for targeted glycoengineering applications where specificity and minimal off‐target effects are critical.

In summary, our findings underscore the importance of integrated evaluation in the development of glycosylation inhibitors and suggest that compounds with limited cell permeability may still exert functional effects through indirect, possibly extracellular or membrane‐associated mechanisms. These insights open new avenues for the design of glycosylation‐modulating tools and contribute to improved strategies for controlling glycoforms in biopharmaceutical production.

## AUTHOR CONTRIBUTIONS

**Matjaž Brinc:** Conceptualization; formal analysis; project administration; supervision; writing – review and editing. **Jaka Kranjc:** Writing – original draft; investigation; methodology; project administration; formal analysis; data curation; visualization. **Marko Anderluh:** Supervision; writing – review and editing. **Stane Pajk:** Writing – original draft; investigation; methodology; formal analysis; data curation; visualization. **Anja Pišlar:** Supervision; writing – review and editing.

## FUNDING INFORMATION

The authors declare that this study received funding from Novartis Pharmaceutical Manufacturing LLC. The funder had the following involvement in the study: design; data collection, analysis, interpretation; writing; and decision to submit for publication.

## CONFLICT OF INTEREST STATEMENT

The authors declare no conflicts of interest.

## Supporting information


**Table S1:** Optimized MS instrument conditions for analyzed compounds.
Figure S1: Viable cell density (VCD) profiles, viability profiles and titers. Cells cultured in deep well plates were supplemented with alendronate at different concentrations (25–200 μM) on day 5. For control water for injection was used at the same final concentration. Red lines and bars represent controls, and different shades of gray alendronate treatment. Standard deviation intervals are indicated by error bar and are mean of three parallel runs.
Figure S2: Impact of B4GALT1 inhibition on glycan group profiles in cell cultures. (A) Relative change of glycan group measurements for selected cell cultures treated with alendronate compared to control. (B) Raw measured values of glycan group measurements. Red bars represent the values measured for control bioprocesses with added water for injection.
Figure S3: Stacked chromatograms of compound 6 showing the standard solution (20 μM) and samples after 15, 30, 60, and 120 min of incubation, arranged from top to bottom.
Figure S4: Stacked chromatograms of compound 8 showing the standard solution (10 μM) and samples after 15, 30, 60, and 120 min of incubation, arranged from top to bottom.
Figure S5: Stacked chromatograms of compound 12 showing the standard solution (50 μM) and samples after 15, 30, 60, and 120 min of incubation, arranged from top to bottom.
Figure S6: Stacked chromatograms of compound 28 showing the standard solution (10 μM) and samples after 15, 30, 60, and 120 min of incubation, arranged from top to bottom.

## Data Availability

The original contributions presented in the study are included in the article and Supplementary material, and further inquiries can be directed to the corresponding author.

## References

[1] Stanley P , Moremen KW , Lewis NE , Taniguchi N , Aebi M . In: Varki A , Cummings RD , Esko JD , et al., eds. Essentials of Glycobiology. Cold Spring Harbor Laboratory Press; 2022. 35536922

[2] Reusch D , Tejada ML . Fc glycans of therapeutic antibodies as critical quality attributes. Glycobiology. 2015;25:1325‐1334. 26263923 10.1093/glycob/cwv065 PMC4634315

[3] van Landuyt L , Lonigro C , Meuris L , Callewaert N . Customized protein glycosylation to improve biopharmaceutical function and targeting. Curr Opin Biotechnol. 2019;60:17‐28. 30554064 10.1016/j.copbio.2018.11.017

[4] Paszkowska J , Kral K , Bieg T , et al. Synthesis and biological evaluation of 5′‐glycyl derivatives of uridine as inhibitors of 1,4‐β‐galactosyltransferase. Bioorg Chem. 2015;58:18‐25. 25462623 10.1016/j.bioorg.2014.11.001

[5] Guo S , Sato T , Shirane K , Furukawa K . Galactosylation of N‐linked oligosaccharides by human β‐1,4‐galactosyltransferases I, II, III, IV, V, and VI expressed in Sf‐9 cells. Glycobiology. 2001;11:813‐820. 11588157 10.1093/glycob/11.10.813

[6] Shauchuk A , Szulc B , Maszczak‐Seneczko D , Wiertelak W , Skurska E , Olczak M . N‐glycosylation of the human β1,4‐galactosyltransferase 4 is crucial for its activity and Golgi localization. Glycoconj J. 2020;37:577‐588. 32827291 10.1007/s10719-020-09941-z PMC7501111

[7] Kuhns S , Shu J , Xiang C , et al. Differential influence on antibody dependent cellular phagocytosis by different glycoforms on therapeutic monoclonal antibodies. J Biotechnol. 2020;317:5‐15. 32361021 10.1016/j.jbiotec.2020.04.017

[8] Raju TS , Jordan RE . Galactosylation variations in marketed therapeutic antibodies. MAbs. 2012;4:385‐391. 22531450 10.4161/mabs.19868 PMC3355481

[9] Zhang Y , Ren S , Sun D , Tao J . Synthesis and evaluation of the analogues for Galactosyl donors as inhibitors of β‐1,4‐galactosyltransferase. Synth Commun. 2010;40:328‐336.

[10] Chung SJ , Takayama S , Wong C‐H . Acceptor substrate‐based selective inhibition of galactosyltransferases. Bioorg Med Chem Lett. 1998;8:3359‐3364. 9873734 10.1016/s0960-894x(98)00618-0

[11] Wang S , Vidal S . Recent design of glycosyltransferase inhibitors. In: Rauter AP , Lindhorst T . eds. Carbohydrate Chemistry: Chemical and Biological Approaches. Vol 39. Royal Society of Chemistry; 2013:78‐101.

[12] Kranjc J , Kramer L , Mikelj M , Anderluh M , Pišlar A , Brinc M . Modulating antibody N‐glycosylation through feed additives using a multi‐tiered approach. Front Bioeng Biotechnol. 2024;12:12. 10.3389/fbioe.2024.1448925 PMC11381414 39253702

[13] Kranjc J , Tomašič T , Pajk S , Brinc M , Pišlar A , Anderluh M . New inhibitors of β‐1,4‐galactosyltransferase I discovered by virtual screening. ChemMedChem. 2025;20:e202400896. 39714887 10.1002/cmdc.202400896 PMC11961147

[14] Agilent Technologies . Agilent AdvanceBio Gly‐X N‐Glycan Prep with InstantPC Kit, 96‐ct (formerly Prozyme). 2023.

