# Supplementary material for: β‐1,4‐galactosyltransferase 1 inhibitors modulate cellular N‐glycan profiles
Source: Biotechnol Prog. 2026 May 21;42(4):e88522. doi: 10.1002/btpr.88522 (PMC13441389; doi:10.1002/btpr.88522)
Supplement: Supplementary file 1 [file BTPR-42-e88522-s001.docx]

*Supporting Information*

β-1,4-galactosyltransferase 1 inhibitors modulate cellular N-glycan profiles

Jaka Kranjc^1^, Stane Pajk^2^, Marko Anderluh^3^, Anja Pišlar^4^, Matjaž Brinc^5^

^1^ Institute for Pharmacy, Faculty of Pharmacy, University of Ljubljana, Aškerčeva cesta 7, SI-1000 Ljubljana, Slovenia

^2^ Department of Pharmaceutical Chemistry, Faculty of Pharmacy, University of Ljubljana, Aškerčeva cesta 7, SI-1000 Ljubljana, Slovenia

^3^ Department of Pharmaceutical Chemistry, Faculty of Pharmacy, University of Ljubljana, Aškerčeva cesta 7, SI-1000 Ljubljana, Slovenia

^4^ Department of Pharmaceutical Biology, Faculty of Pharmacy, University of Ljubljana, Aškerčeva cesta 7, SI-1000 Ljubljana, Slovenia

^5^ Bioprocess development, Technical Development Biologics, Novartis Technical Research & Development, Novartis LLC, Kolodvorska 27, SI-1234 Mengeš, Slovenia

**Table S1**: Optimized MS instrument conditions for analyzed compounds.

|  | Compound **6** | Compound **8** | Compound **12** | Compound **28** |
| --- | --- | --- | --- | --- |
| Injection volume (µL) | 5 | 25 | 10 | 10 |
| Retention time (min) | 3.30 | 3.24 | 2.73 | 3.03 |
| Precursor ion (m/z) | 546.14 | 284.09 | 191.03 | 308.05 |
| Product ion (m/z) | 212.06 | 152.05 | 91, 121, 147 | 205 |
| Collision energy (V) | 25 | 20 | 30 | 25 |
| Collision-induced dissociation gas (mTorr) | 2 | 2 | 2.5 | 2 |
| Dwell time (ms) | 100 | 300 | 300 | 100 |
| Polarity | Positive | Positive | Negative | Positive |
| Ion source voltage (V) | 3500 | 3500 | 4000 | 3500 |
| Sheath gas (AU) | 50 | 60 | 40 | 50 |
| Auxiliary gas (AU) | 10 | 12 | 10 | 10 |
| Sweep gas (AU) | 1 | 1 | 1 | 1 |
| Vaporizer temperature (°C) | 80 | 200 | 200 | 140 |

**Figure S1**: Viable cell density (VCD) profiles, viability profiles and titers. Cells cultured in deep well plates were supplemented with alendronate at different concentrations (25 – 200 μM) on day 5. For control water for injection was used at the same final concentration. Red lines and bars represent controls, and different shades of grey alendronate treatment. Standard deviation intervals are indicated by error bar and are mean of three parallel runs.

**Figure S2**: Impact of B4GALT1 inhibition on glycan group profiles in cell cultures. A) Relative change of glycan group measurements for selected cell cultures treated with alendronate compared to control. B) Raw measured values of glycan group measurements. Red bars represent the values measured for control bioprocesses with added water for injection.

**Figure S3**: Stacked chromatograms of compound **6** showing the standard solution (20 µM) and samples after 15, 30, 60, and 120 min of incubation, arranged from top to bottom.

**Figure S4**: Stacked chromatograms of compound **8** showing the standard solution (10 µM) and samples after 15, 30, 60, and 120 min of incubation, arranged from top to bottom.

**Figure S5**: Stacked chromatograms of compound **12** showing the standard solution (50 µM) and samples after 15, 30, 60, and 120 min of incubation, arranged from top to bottom.

**Figure S6**: Stacked chromatograms of compound **28** showing the standard solution (10 µM) and samples after 15, 30, 60, and 120 min of incubation, arranged from top to bottom.
